# MicroRNA expression profiles differ between primary myofiber of lean and obese pig breeds

**DOI:** 10.1371/journal.pone.0181897

**Published:** 2017-07-31

**Authors:** Dongting He, Tiande Zou, Xiangrong Gai, Jideng Ma, Mingzhou Li, Zhiqing Huang, Daiwen Chen

**Affiliations:** 1 Institute of Animal Nutrition, Sichuan Agricultural University, Chendu, Sichuan, People’s Republic of China; 2 State Key Laboratory of Animal Nutrition, Ministry of Agriculture Feed Industry Centre, China Agricultural University, Beijing, People’s Republic of China; 3 Institute of Animal Genetics and Breeding, Sichuan Agricultural University, Chendu, Sichuan, People’s Republic of China; Wageningen UR Livestock Research, NETHERLANDS

## Abstract

MicroRNAs (miRNAs) are non-coding small miRNAs ~22 nucleotides in length and play a vital role in muscle development by binding to messenger RNAs (mRNAs). Large White (LW, a lean type pig) and Meishan pigs (MS, a Chinese indigenous obese breed) have significant postnatal phenotype differences in growth rate, muscle mass and meat quality, and these differences are programmed during prenatal muscle development. Little research shed light directly on the miRNA transcriptome difference in prenatal muscles between these two distinct pig breeds. Myofiber phenotypes of LW and MS were measured at developmental stages of 35, 55 and 90 days post-conception (dpc), which revealed that the myogenesis process is more intense in MS than in LW at 35 dpc. To investigate the role of miRNAs involved in regulating muscle development at earlier stages of myogenesis and decipher the miRNAs transcriptome difference between LW and MS, here, the miRNAomes of *longissimus dorsi* muscle collected at 35 dpc from female LW and MS were analyzed by deep sequencing. Overall, 1147 unique miRNAs comprising 434 known miRNAs, 239 conserved miRNAs and 474 candidate miRNAs were identified. Expression analysis of the 10 most abundant miRNAs in every library indicated that functional miRNAome may be a small amount and tend to be greater expressed. These sets of miRNA may play house keeping roles that were involved in myogenesis. A total of 87 miRNAs were significantly differentially expressed between LW and MS (reads > 1000, *P* < 0.05). Gene ontology (GO) and KEGG pathway enrichment analysis revealed that the differentially expressed miRNAs (DE miRNAs) were associated mainly with muscle contraction, WNT, mTOR, and MAPK signaling pathways. Some myogenesis related miRNAs (miR-133, miR-1, miR-206 and miR-148a) are highly abundant in MS, while other miRNAs (let-7 family, miR-214, miR-181) highly expressed in LW. In addition, the expression patterns of miRNAs (miR-1, -133, -206) at three prenatal stages (35, 55 and 90 dpc) were determined using qRT-PCR. Notably, ssc-miR-133 was significantly more highly expressed in LW pigs skeletal muscle at all prenatal stages compared with its expression in LW pigs skeletal muscle. Taken together, the main functional miRNAs during muscle development are different between lean and obese pig breeds. The present study adds new information to existing data on porcine miRNAs and will be helpful to investigate the dominant (main functional) muscle-related miRNAs sets in different pig breeds.

## Introduction

Lean pig breeds, such as Large White (LW), have been intensively selected over the past decades for improved growth rate and muscularity, which is believed to have led to deterioration in meat quality [1]. The obese pig breeds, such as Meishan (MS) pigs, exhibit lower growth rate, poor feed efficiency and lower lean meat content, but sensory quality of their meat is superior [2], mainly because of relatively greater intramuscular fat content and reddish meat color [3].

Both total number of fibers (TNF) and fiber types are related to meat quality. In pigs, types I, IIa, IIx and IIb myosin heavy chain (MyHC) are expressed in skeletal muscle [1]. The percentage of type IIb fiber was negatively correlated to postmortem muscle PH, color and drip loss [4]. The type I fiber has been reported to improve tenderness [5]. Muscle with more type I and IIa fibers show greater intramuscular fat content and reddish color [6]. In *longissimus dorsi* muscle, IIa and IIx fibers were elevated in MS pigs, whereas the IIb fiber was more highly expressed in LW pigs [3]. High TNF results in increasing muscle fiber size, which influence meat quality negatively in drip loss and tenderness [7, 8]. The TNF is fixed in porcine fetus period, and postnatal muscle growth mainly depends on myofiber hypertrophy. The TNF results from two successive generations of myofibers, i.e. primary generation forms from 35 until 55 days post-conception (dpc), followed by a secondary generation which forms between 55 and 90–95 dpc. These secondary fibers form around the primary myotubes, using them as a scaffold [9, 10]. Thus, the number and size of primary fibers have a positive correlation with the total number of secondary fibers and TNF. Previous studies have shown that the TNF was found to be dramatically lower in MS than LW pigs at birth [2]. The above analysis indicate that the differential meat quality between LW and MS pig breeds already starts to develop during early prenatal development.

MicroRNAs (miRNAs) are small (~22 nucleotides) non-coding RNAs that regulate gene expression at the post-transcriptional level via translational inhibition or mRNA degredation. Emerging evidence has demonstrate that miRNAs play a critical role in skeletal muscle biology. For example, miR-133 enhances myoblast proliferation by repressing serum response factor (SRF). By contrast, miR-1 promotes muscle differentiation by targeting histone deacetylase 4 (HDAC4) [11]. MiR-206 pushes the equilibrium toward differentiation by down-regulating Id1-3 and MyoR [12]. In addition, increasing evidence has shown a dynamic change in miRNA expression during developmental stages of the porcine skeletal muscle, e.g., miR-133 was up regulated from 65 to 90 days of gestation [13], and miR-1a and miR-133a showed highest abundance during the fast growing stage at postpartum day 120 [14]. Nonetheless, little is known about the difference in miRNAs expression profiles in skeletal muscle between LW and MS during prenatal development.

Here, we hypothesized that there might be some difference between lean pig breeds (LW) and obese pig breeds (MS) during embryonic skeletal muscle development, which may be related to their divergence in meat quality. The phenotypic properties of skeletal muscle were detected at developmental stages 35-, 55-, 90-day old fetuses for LW and MS pig breeds. To explore the contribution of miRNAs to muscle phenotype variance in primary myofibers (fibers at 35 dpc) of LW and MS, differentially expressed miRNAs (DE miRNAs) of primary myofibers between the two pig breeds were investigated using a deep sequencing approach. The biological functional analysis of DE miRNAs was conducted. Finally, the expression patterns of muscle specific miRNA (*i*.*e*., miR-1, miR-133 and miR-206) were detected in LW and MS. Our study extended the repertoire and understanding the roles of miRNAs in muscle development, thus help us to further understand the molecular mechanisms responsible for breed-specific differences in growth performance and meat quality.

## Materials and methods

### Ethics statement

All research involving animals were conducted according to the Regulations for the Administration of Affairs Concerning Experimental Animals (Ministry of Science and Technology, China, revised in June 2004) and approved by the Animal Care and Use Committee of Sichuan Agricultural University under permit number DKY-B20131704. The fetuses used in this study were carbon dioxide asphyxiation for euthanasia.

### Animals and sample collection

A total of 12 LW gilts (average body weigh = 135.54 ± 3.27kg) and 12 MS gilts (average body weigh = 71.8 ± 2.75kg) were mated to boars of the same breed (LW or MS). From gestation, sows were fed the diet with digestible energy (DE) of 3000 cal/kg, they were individually fed 2.0 kg/d until 35 days post-conception (dpc), then 2.4 kg/d until 90 dpc and 3.0 kg/d until delivery. The average period of study was 120 days. At each prenatal stage of 35-, 55- and 90 dpc four sows per breed were anesthetized (Zoletil 50, Virbac; 4mg/kg body weigh) for cesarean. The *longissimus dorsi* (LD) muscle tissues were isolated from fetus, and immediately frozen in liquid nitrogen and stored at -80°C. The muscle tissues used for frozen section which were immersed in liquid nitrogen until slice.

### Histologic analyses

Muscle tissues were taken out of liquid nitrogen. Frozen sections (8μm) were cut in a cryostat (-20°C) on microscope slides (Ploysine Adhesion Slide; Head Biotechnology, China, Beijing). Slides were allowed to dry in the air and stain with haematoxylin and eosin (H&E). At each prenatal stages of 35, 55 and 90 dpc, frozen sections were made from four fetus of different sows. Four slides were made per fetus and images were captured under the inverted microscope (Nikon Eclipse TS100, Nikon, Tokyo, Japan) at 400× magnification. Five random fields were analyzed per section to determine the density (number of myofiber per mm^2^) of muscle fibers. About 200 fibers per fetus were evaluated to determine diameters of myofiber. All datas were measured with Image-Pro Plus 6.0.

### Small RNA library preparation and sequencing

For small RNA library construction, three muscle total RNA samples were isolated from the 35 dpc fetuses per breed using mirVana^™^ miRNA isolation kit (Ambion, Austin, USA). For each breed, three female fetus from different sows were selected according to consistent placenta location. Sex determination of the 35 dpc fetuses was performed using a PCR amplification of a sex-determining region on the Y chromosome as described by Li *et al*. [15] (S1 Fig). The total RNA isolated from LW and MS generated six small RNA libraries: LW1, LW2, LW3, MS1, MS2 and MS3. The total RNA quality and purity were measured using a Bioanalyzer 2100 and RNA 6000 Nano LabChip Kit (Agilent, CA, USA) with RIN > 7.0. Approximately 1 μg total RNA per sample was used to prepare small RNA library according to protocol of TruSeq Small RNA Sample Prep Kits (Illumina, San Diego, USA). In general, the processing consisted of the following successive steps: 10~40 nt RNA fragment were excised, purified from a polyacrylamide gel electrophoresis (PAGE), and ligated with 5’ and 3’ adaptors using T4 RNA ligase. Then the modified small RNA was reverse transcripted and amplificated by RT-PCR. Subsequently, the amplified cDNA constructs were purified from agarose gel. Finally the enriched cDNA was quantified in an Bioanalyzer 2100 (Agilent, CA, USA) and sequenced in a HiSeq 2500 sequencing system (Illumina, San Diego, USA) at the LC-BIO (Hangzhou, China).

### Sequencing data analysis

The raw reads were subjected to the Illunina pipeline filter (Solexa 0.3), and then the dataset was further processed with an in-house program, ACGT101-miR (LC Science, Houston, Texas, USA) to remove sequencing adapters, junk reads and fragments < 18 nt and > 26 nt. Subsequently, the remaining 18~26 nt reads were searched against the Rfam, NCBI and Repbase database to remove non-miRNAs (i.e. rRNA, tRNA, snRNA, snoRNA, mRNA and repeats). Those sequenced reads survived from above strict filter rules were deemed to “mappable reads” or high quality reads and used for further analysis.

### Identification of porcine miRNA

The mappable reads were mapped to the pig genome (Sscrofa 10.2) using NCBI Local BLAST, including three steps: (1) the mappable reads were aligned to porcine pre-miRNAs/miRNAs, and then to pre-miRNAs/miRNAs from 25 other mammals in miRBase 21.0; (2) the mapped pre-miRNAs /miRNAs in step1 were searched against the pig genome to determine their genomic locations and annotations in Ensemble (Sscrofa 10.2); (3)the unmapped sequences were further blasted against the pig genomes, and the hairpin RNA structures containing sequences were predicated from the flank 80 nt sequences using RNAfold software. After the above analysis, three kinds of porcine miRNAs were identified. First, reads map to specific miRNAs/pre-miRNAs in miRbase and the pre-miRNAs further map to the pig genome & EST, were defined as porcine known miRNAs, marked with “ssc-miR-”. Second, the reads map to other mammalian miRNAs/pre-miRNAs in miRbase and the pre-miRNAs further map to the pig genome & EST, were defined as porcine conserved miRNAs, marked with “PC-”. Third, there are two kinds of reads: (1) the reads can map to selected miRNAs/pre-miRNAs in miRbase. The mapped pre-miRNAs do not map to the genome, but the reads (and of course the miRNAs of the pre-miRNAs) map to genome. The extended genome sequences from the genome loci may form hairpins. (2) The reads do not map to selected pre-miRNAs in miRbase. But the reads map to genome & the extended genome sequences from genome may form hairpins. The above two were defined as porcine candidate miRNAs, they were also predicted novel miRNAs for pig, so marked as “PN-”. These identified porcine miRNAs were assembled into porcine unique miRNAs according to the unique miRNA sequence. In the above tables, the expression of miRNAs in six libraries were normalized by total mappable reads, and used for further analysis.

### Differentially expressed miRNA analysis

The differentially expressed miRNAs were identified based on the normalized most abundant sequence reads. The normalized miRNA reads, > 1000 reads counts in either of LW and MS, from the two breeds were compared using Student *t*-test. The significance threshold was set to be 0.05 in *t*-test. After that the top 50 most abundant DE miRNAs, according to the total reads counts of LW and MS, were choosed to perform the Go and KEGG pathway analysis.

### Functional analysis of miRNAs

To predict the genes targeted by most abundant miRNAs and differentially expressed miRNAs, two computational target prediction algorithms (TargetScan 6.2 and miRanda) [16, 17] were used to identify miRNA biding site. Finally, the data predicted by both algorithms were combined and the overlaps were calculated. As porcine genes were not included in the current version of the above-mentioned algorithms, prediction was performed using human miRNAs. The Gene Ontology (GO) terms and Kyoto Encyclopedia of Genes and Genomes (KEGG) Pathway of these miRNA targets were annotated using DAVID bioinformatics resources (http://david.abcc.ncifcrf.gov/) [18]. The small RNA sequence data and processed files have been submitted to NCBI Gene Expression Omnibus (GEO) under accession No. GSE88860.

### RT-qPCR and statistics analysis

Total RNA was extracted using RNAiso Plus reagent (TaKaRa, China). RNA concentration was measured using NanoVue Plus (GE, US). The A260/A280 ratios of all samples ranged from 1.8 to 2.0, which indicate the samples were of good quality. RNA integrity was further checked by agarose gel electrophoresis. RT-qPCR (quantitative Real-time PCR) for mRNA was performed as previously described [19]. Six myogenesis related genes (*Pax7*, *MyoD*, *Myf5*, *MyoG*, *MRF4*, *CKM*) were evaluated in this study. The primer sequences are available in S1 Table. The miRNAs levels were quantified using the S-poly(T) miRNA qPCR-assay method as described [20] using the reagent kit (Geneups, China). SnoRNA-202, a commonly used mouse internal reference, was used as the internal control for normalization. Mus snoRNA-202 and sus scrofa snoRNA-68 shared high sequence similarity when subjected to NCBI BlAST analysis, and Ct values of snoRNA-202 was consistent and had little variation from sample to sample. All of the data are expressed as the mean ± SD, or error bars depict SD. The 2^-ΔΔCt^ method was used to analyze real-time PCR data. Expression of mRNA or miRNA was presented as the fold of the mean of LW group. The difference of genes (*Pax7*, *MyoD*, *Myf5*, *MyoG*, *MRF4*, *CKM*) was analyzed through one-way ANOVA model followed by Duncan’s multiple range analysis using IBM SPSS Statistics 19 (IBM SPSS Inc., Chicago, IL, USA). The difference of miRNAs (miR-133, miR-1 and miR-206) among six samples (the muscle of LW and MS fetus at each time point 35d, 55d and 90d) was analyzed through two-way ANOVA method, in which breeds and time points were factors. For the validation of sequenced result, the Pearson correlation coefficient (r) and corresponding significance value (*P*) was also calculated by SPSS software. Each fetus was considered as an experimental unit. Statistical significance was defined as *P* < 0.05.

## Results and discussion

### Primary myofiber morphology and transcriptome data

**Fig 1:**
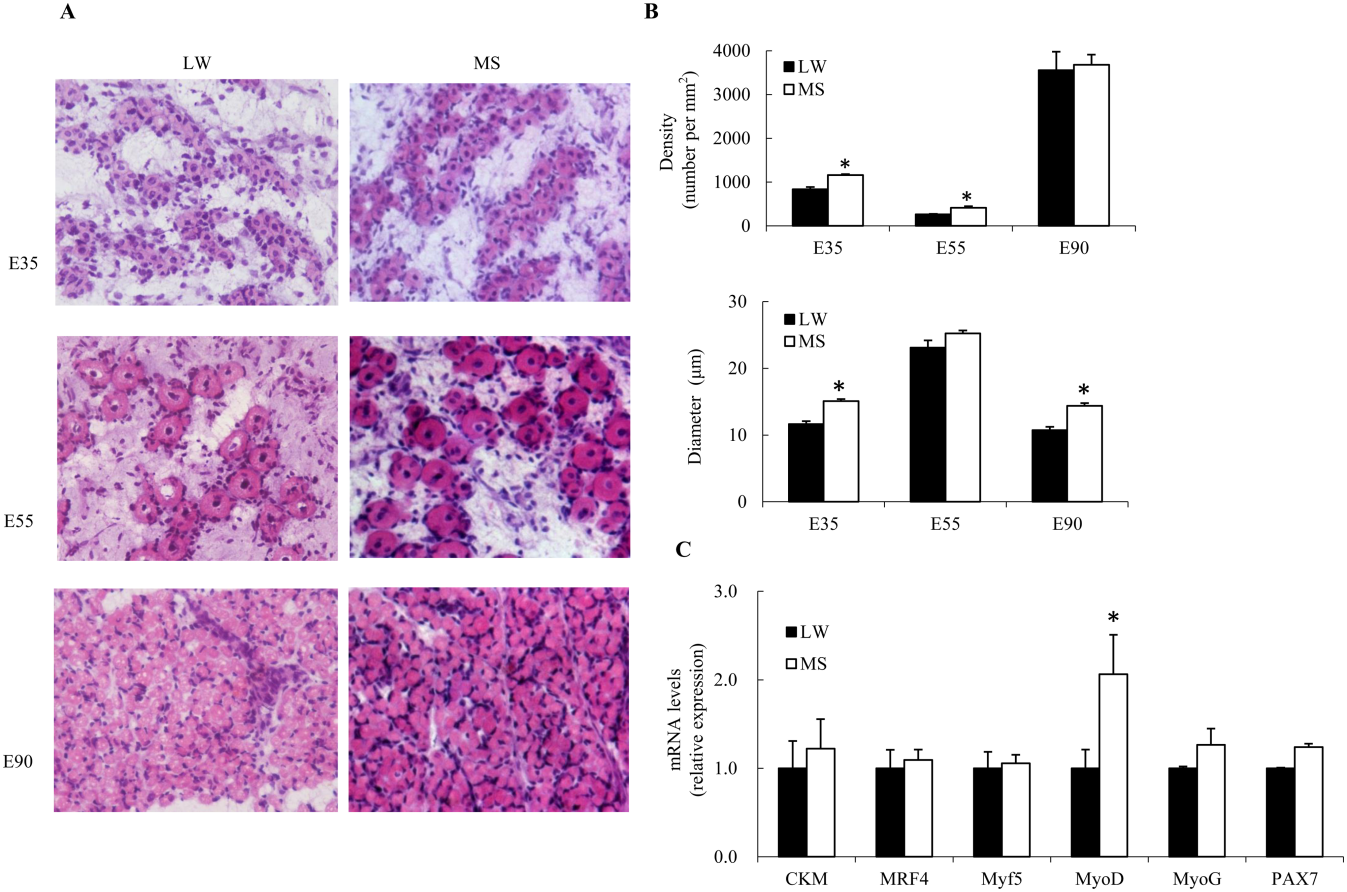
**Comparison of myofiber density and diameter between Large White (LW) and Meishan (MS) fetuses (A, B) during skeletal muscle development at 35 to 90 dpc (E35, E55 and E90). The mRNA expression of Myogenesis-related genes in *longissimus dorsi* between LW and MS at 35 dpc (C).** Values are mean ± SD, *n* = 4. *Mean values were significantly different, *P*<0.05.

To study whether the myogenesis process was different between LW and MS pigs during embryonic period, myofiber density and diameter were determined for porcine embryo in the 35, 55 and 90 dpc, respectively. As shown in Fig 1A and 1B, the myofiber density and diameter of 35 dpc were significantly higher in MS compared to LW (*P*<0.05), which indicated the molecular mechanisms of primary myogenesis differ between these two breeds. In addition, expression of myogenesis related genes were detected in the 35 dpc skeletal muscles. The mRNA expression of *MRF4*, *Myf5*, *MyoD*, *MyoG*, *CKM*, and *Pax7* genes were greater in MS than in LW, especially the expression level of *MyoD* (*P* < 0.05) (Fig 1C). The genes *MyoD*, *Myf5*, *MyoG*, *MRF4* belong to the MRF gene family taking a central position in the regulation of myogenesis [21]. The CKM, muscle-creatine kinase, play a crucial role in energy metabolism. A high creatine kinase level, suggestive of a high energy level, was reported to be associated with hypertrophic growth [22]. Pax7 is a transcription factor that plays a role in myogenesis through regulation of muscle precursor cells proliferation [23]. Taken together, all these results suggest that the myogenesis process of primary myofiber is more intense in MS than in LW.

### Overview of the high-throughput sequencing data

Previous studies revealed that miRNAs play more important roles at earlier stages of myogenesis than at later stages [24]. To explore the contribution of miRNAs to myogenesis in primary myofiber of LW and MS, a deep sequencing approach was applied. The sequencing of small RNA libraries from LW and MS at 35 dpc yielded 6.93 ± 1.60 Million (M) and 9.24 ± 3.70 M raw reads, respectively. After filtering different classes of known small RNAs, a total of 4.09 ± 0.94 M and 4.64 ± 1.23 M mappable reads were retained for LW and MS, respectively (S2 Fig), which were then deemed as miRNA candidates. The length of mappable sequences ranged from 21~23 nt with a distribution peak at 22 nt, which was consistent with the common size of miRNAs (S3 Fig).

**Fig 2:**
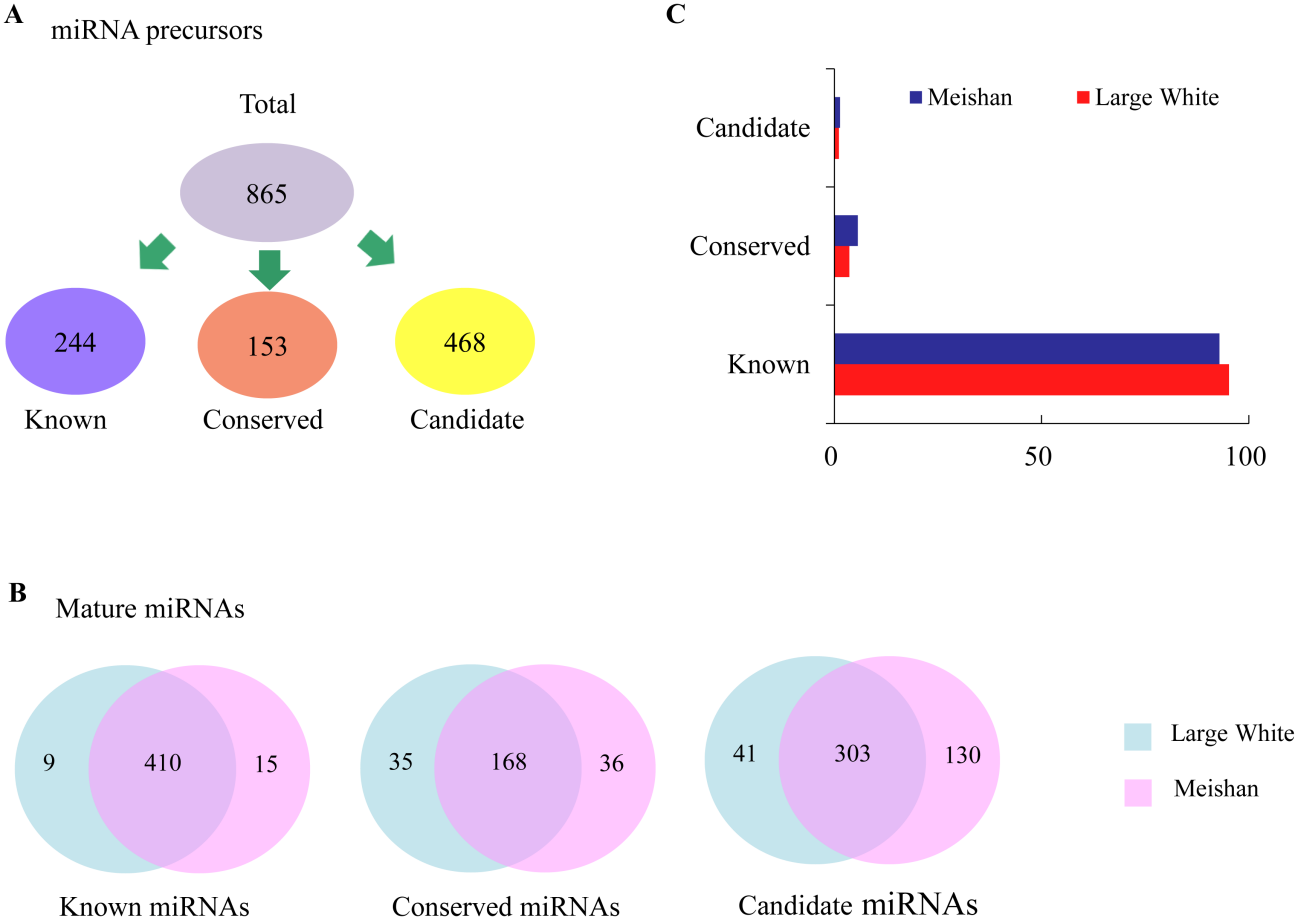
Description of miRNAs in two pig breeds. (A) Overview of the miRNA precursors including known, conserved and candidate miRNAs between Large White (LW) and Meishan (MS). (B) Venn charts indicate expression patterns of known, conserved and candidate miRNAs. (C) Distribution of read counts between LW and MS.

After blasting these mappable reads against pig genome, miRBase (21.0), and predicting the hairpin structures, in total, 865 pre-miRNAs encoding 1147 mature miRNAs in six sequencing libraries were identified, including 434 known miRNAs, 239 conserved miRNAs and 474 candidate miRNAs (Fig 2A; S2 and S3 Tables). Most miRNAs were co-expressed in LW and MS and account for a large portion (Fig 2B), while the miRNAs that specifically expressed in one breed were in low reads abundance (< 20 reads) (S2 and S3 Tables). Of the 1147 unique miRNAs, 37.84% (434/1147) were porcine known miRNAs, which accounted for 92.83% of the total sequence reads. However, the sequence reads of conserved miRNAs and candidate miRNAs represented only a relatively small fraction (5.66%, 1.50%) of the total sequence reads (Fig 2C). These results suggested the high-confidence of our sequencing data.

**Fig 3:**
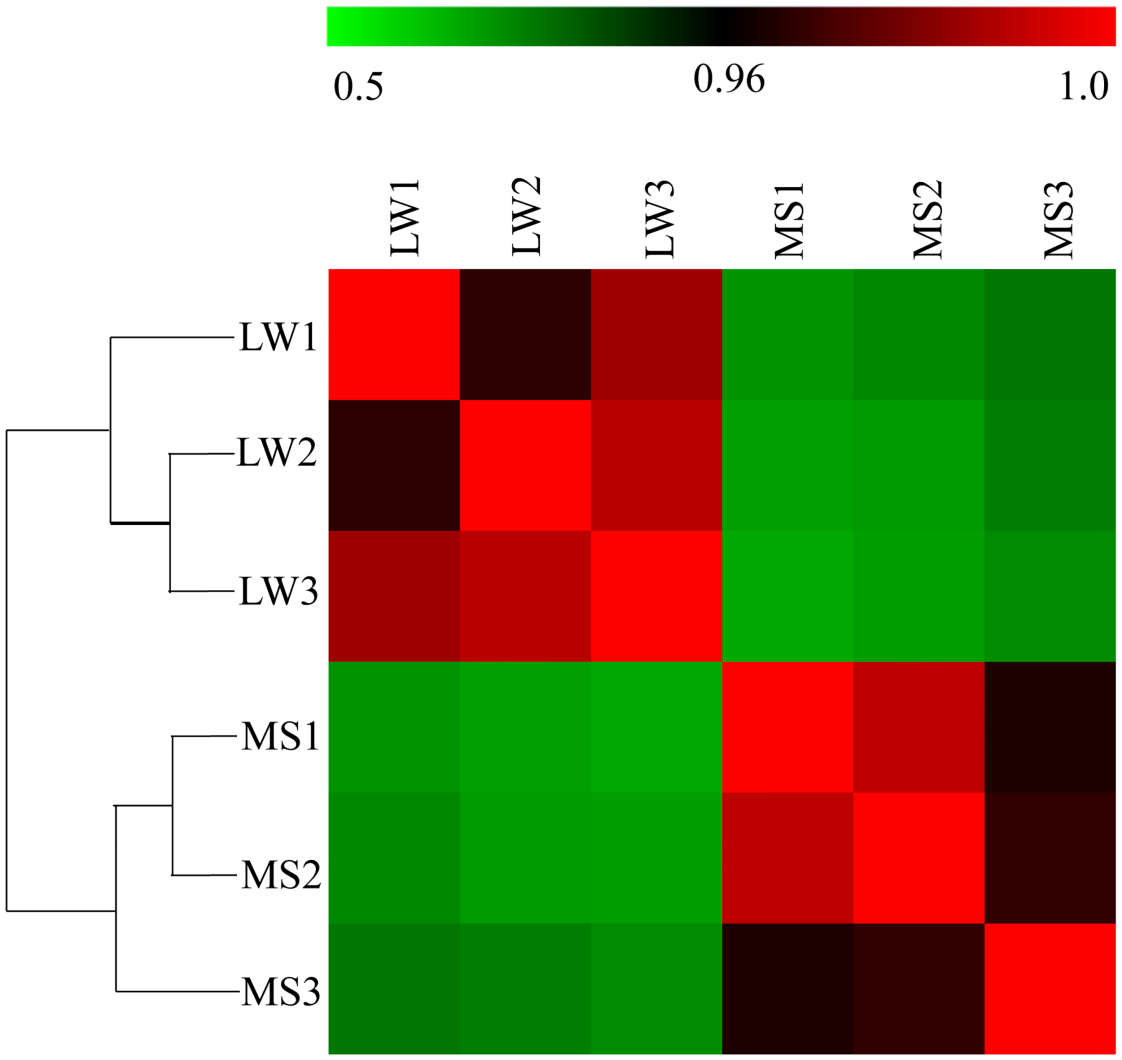
Hierarchical clustering analysis and heat map matrix of Pearson correlations of the reads counts of 1147 unique miRNAs between Large White (LW) and Meishan (MS). LW1, LW2 and LW3 refer to the three biological replications of Large White pigs; MS1, MS2 and MS3 refer to the three biological replications of Meishan pigs. The color legend at top represents the correlation coefficient.

Furthermore, to further characterize variability in miRNA expression profiling, a hierarchical clustering analysis was performed based on the expression of 1147 unique miRNAs. As shown in Fig 3, the miRNA expression profiles showed two major clusters: one is the three biological replicates of LW, and one is the three biological replicates of MS. Meanwhile, three biological replicates of LW and MS were highly correlated (LW: average *r* = 0.9803; MS: average *r* = 0.9743), which indicates highly experimental reliability and good reproducibility of replicates (Fig 3).

### Analysis of highly abundant miRNAs

**Fig 4:**
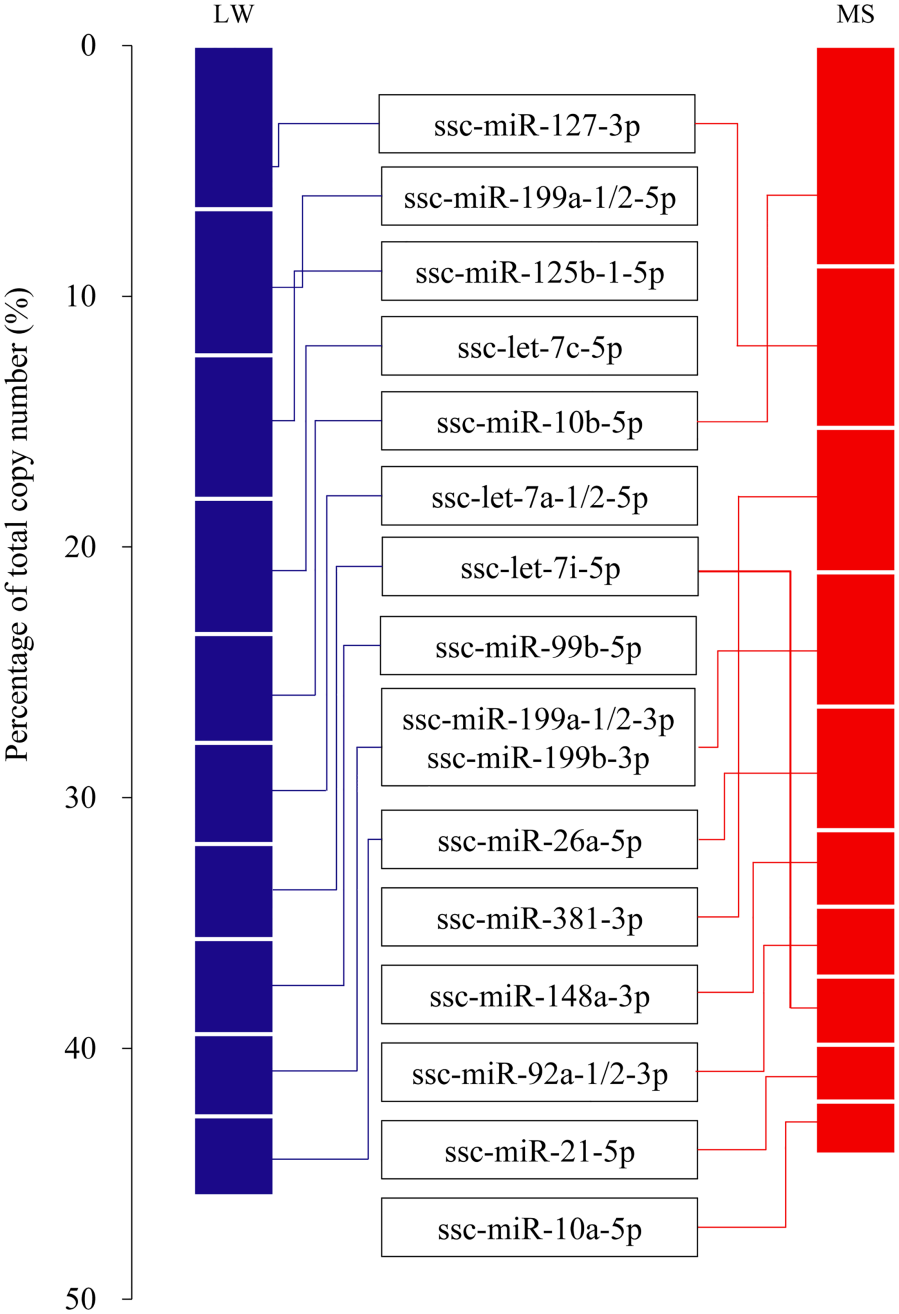
Top 10 unique miRNAs with the highest reads counts.

Expression analysis of the 10 most abundant miRNAs in each library indicated that functional miRNAome may be confined and tend to be highly expressed. As shown in Fig 4, the top 10 miRNAs with the highest abundance contributed 45.89% and 44.23% of the total counts in the LW and MS libraries, respectively. The unified set of top 10 unique miRNAs over the two pig breeds correspond to 15 unique miRNAs, 11 of which (ssc-let-7a-1/2-5p, ssc-let-7c-5p, ssc-let-7e-5p, ssc-miR-10a-5p, ssc-miR-10b-5p, ssc-miR-127-3p, ssc-miR-148a-3p, ssc-miR-199a-1/2-5p, ssc-miR-21-5p, ssc-miR-26a-5p, ssc-miR-125b-1-5p) had been frequently reported highly expressed in skeletal muscle during porcine prenatal and postnatal developmental stages. For example, Qin et al. had reported that ssc-let-7a, ssc-miR-10a, ssc-miR-10b, ssc-miR-127, ssc-miR-148a, ssc-miR-21, ssc-miR-26a were the most abundant miRNAs during porcine skeletal muscle developmental stages from 35 days post coitum to postnatal day 180 [25]. Hou et al. shows that ssc-let-7a, ssc-let-7c and ssc-miR-26a were the common most abundant miRNAs in longissimus dorsi muscle of three pig breeds (Landrace, Tongcheng, and Wuzhishan) at postnatal day 240 [26]. Study of Mai et al. revealed that ssc-let-7a-1/2-5p, ssc-miR-10a-5p, ssc-miR-127-3p, ssc-miR-148a-3p, ssc-miR-199a-1/2-5p, ssc-miR-26a-5p were the most highly expressed unique miRNAs over five porcine muscle developmental stages from 90 dpc to 7 y after birth [27].

In the unified 15 miRNAs set, ten of which have the highest abundance in all two breeds (Fig 4). These ten miRNAs may have housekeeping cellular roles and may be the main regulatory miRNAs in myogenesis. For example, miR-199a was reported to regulate cell proliferation by targeting *Dyrk1a* through the Calcineurin/NFAT pathway [28]. MiR-125b negatively modulates myoblast differentiation in culture and muscle regeneration in mice by targeting *IGF-II* [29]. Let-7a, let-7c and let-7i (members of let-7 family) are regulators in development, and in cellular basal metabolism [30]. MiR-26a is up-regulated during myoblast differentiation and can suppress Enhancer of Zeste homolog 2 (*EZH2*) to promote myogenesis [31]. In addition, miR-127, miR-99b and miR-10b are important regulators in various cancers [32–34].

Interestingly, the TOP 30 most abundant miRNAs of LW embryo were similar to those of MS embryo (S2 Table). But among their common TOP 30 miRNAs from the total reads, 17 miRNAs were differentially expressed between LW and MS. Above results may suggest that whatever the pig breeds is, the highly expressed miRNAs profiles were similar at a certain development stage, but the expression levels of miRNAs varies among different pig breeds.

### Differentially expressed miRNAs and functional analysis

**Fig 5:**
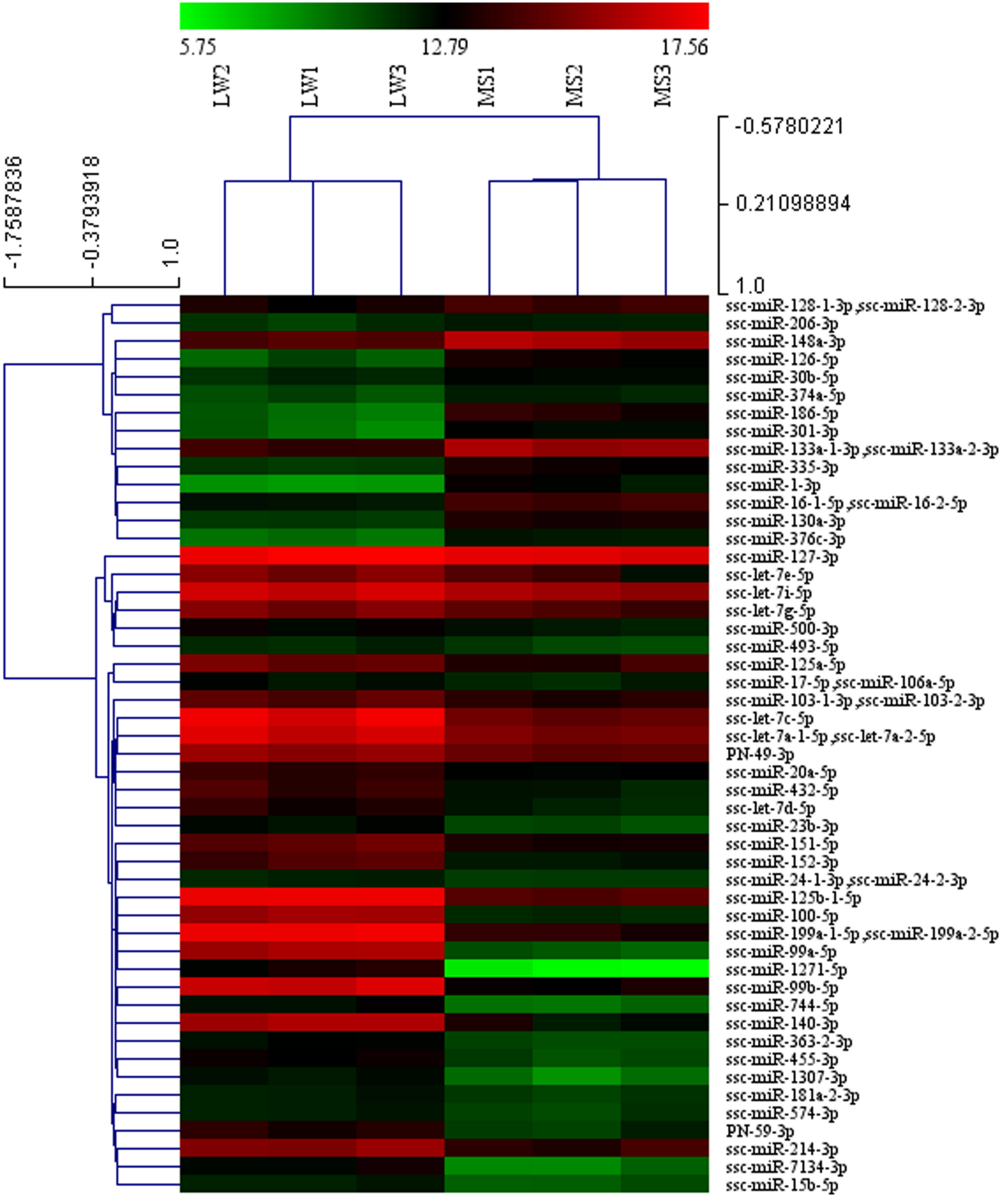
Hierarchical cluster analysis of the top 50 differentially expressed miRNAs (*P* < 0.05). Normalized log (base 2) data was hierarchically clustered by miRNA reads counts and is plotted as a heat map. LW1, LW2 and LW3 refer to the three biological replications of Large White pigs; MS1, MS2 and MS3 refer to the three biological replications of Meishan pigs.

**Fig 6:**
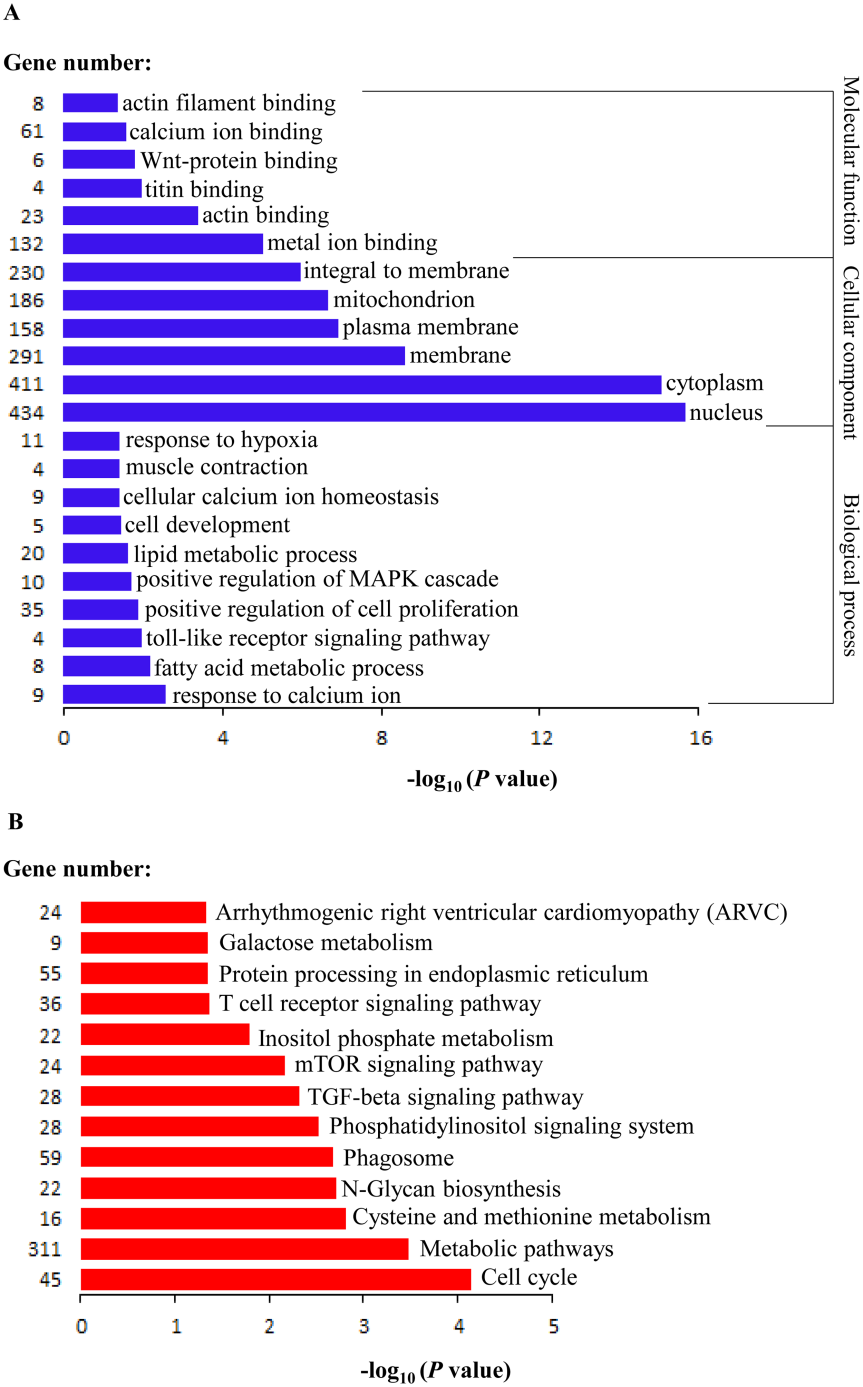
GO (A) and KEGG (B) pathways analysis of top 50 differentially expressed miRNAs.

We identified 87 differentially expressed miRNAs between LW and MS pigs (with miRNAs read counts >1000, *p*-value of t-test < 0.05), of which 29 were upregulated and 58 were downregulated in MS pigs (S4 Fig, S4 Table). Interestingly, among the differentially expressed miRNAs (DE miRNAs) library, the top 50 most abundant miRNAs account for 95.87% of the total DE miRNAs reads, indicating their important roles in myogenesis. Subsequently, the top 50 DE miRNAs were subjected to a hierarchical analysis, and the result showed that these genes were gathered into two major clusters: one is 14 upregulated miRNAs, and one is 36 down regulated miRNAs (Fig 5). In total, the target genes of 14 upregulated miRNAs significantly enriched in 332 GO terms and 45 KEGG pathways (S5 Table) (*P* < 0.05). In addition, the target genes of 36 downregulated miRNAs significantly enriched in 325 GO terms and 53 KEGG pathways (S5 Table) (*P* < 0.05). The 14 upregulated miRNAs and 36 downregulated miRNAs have 199 GO terms and 32 KEGG pathways in common (S5 Table), of which some significantly enriched GO terms and KEGG pathways were picked out to illustrate the possible roles of DE miRNAs during muscle development (Fig 6). Particularly, the DE miRNAs were mainly involved in the regulation of muscle functions such as actin filament binding, calcium ion binding, actin binding, muscle contraction, cellular calcium ion homeostasis and response to calcium ion. Furthermore, DE miRNAs were intensively involved in not only the metabolic pathways such as lipid metabolic process, fatty acid metabolic process, galactose metabolism, cysteine and methionine metabolism but also signaling pathways such as wnt-protein binding, toll-like receptor, mTOR, TGF-β, positive regulation of MAPK cascade signaling pathways (Fig 6). Many studies have reported that the TGF-β family played important roles in skeletal muscle development [35, 36]. MAPK signalling pathway is known to be involved in regulation of muscle differentiation by affecting the activities of myogenic transcription factors as well as controlling the expression of structural muscle genes [37]. Wnt signaling pathway involved in embryonic myogenesis and in regulating the homeostasis of adult muscle, resulting its universal enrichment from embryonic to adult myofiber maturation [38]. Calcium signaling was considered potential mediators of postnatal muscle development and hypertrophy [39]. These analyses illustrate some possible reasons for different myogenesis potential between LW and MS.

In top 50 DE miRNAs, many of them were previously reported to associated with skeletal muscle development. For example, miR-1 directly targets HDAC4, a transcriptional repressor of muscle gene expression to promote myogenesis, and miR-133 enhance myoblast proliferation by repressing serum factor (SRF) [40]. MiR-206 can be upregulated by MyoD and MEF2, and can promote the differentiation of myoblast by targeting multiple genes, such as Id1-3, Pax 7, BDNF, Notch-3, Hmyb3 and Cx43 [41, 42]. MiR-148a mediated myogenic differentiation via targeting ROCK1 [43]. MiR-214 may be related to embryonic myogenesis [44] and regulate both proliferation and differentiation of myoblasts depending on the conditions [45]. MiR-24 has been linked to the inhibition of skeletal muscle differentiation by transforming TGF-β [46]. The microRNA miR-181 can target the homeobox protein Hox-A11 in early stage of myoblast differentiation [47]. MiR-17 and miR-20a belong to the miR-17-92 family which regulates cell proliferation and collagen synthesis by targeting TGF-β pathway [48]. MiR-126 attenuated insulin signaling [49] and governed vascular integrity and angiogenesis [50], suggesting their interactions with signaling pathways were required for muscle normal development and maintenance. Interestingly, Among them, miR-133, miR-1, miR-206 and miR-148a were highly abundant in MS pigs, while let-7 family, miR-214 and miR-181 were highly expressed in LW. These results indicated that the main functional miRNAs during muscle development are different between lean and obese pig breeds.

Interestingly, the highly abundant DE miRNA PN-49-3p (reads counts > 10000) may be a novel porcine miRNA participating in embryonic skeletal development. In our study, the GO category of the PN-49-3p was enriched in nucleus, zinc ion binding, embryonic placenta development and cellular response to corticotropin-releasing hormo et al. (S5 Fig). The KEGG pathway enriched in endocytosis, phagosome, steroid hormone biosynthesis, T cell receptor signaling pathway and bacterial invasion of epithelial cells et al. (S6 Fig). The above analysis indicates that PN-49-3p may participant in biological process of immune and anti-inflammatory. And the role of PN-49-3p in skeletal muscle prenatal development process need further studies.

**Fig 7:**
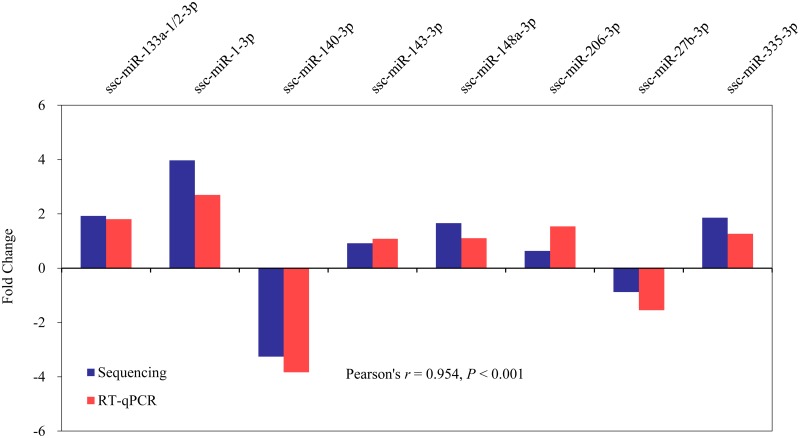
Validation of sequenced results using RT-qPCR for eight representative DE miRNAs. The RT-qPCR result for miRNAs fold change (FC) between LW and MS was calculated as FC = 2^-ΔCt MS^ /2^-ΔCt LW^, and the sequenced result for miRNA log_2_FC expression levels was calculated by log_2_FC = log_2_(reads of LW/ reads of MS), The sequenced reads were normalized.

Furthermore, the expression levels of the 8 differentially expressed miRNAs show good correlation (Pearson’s *r* = 0.954, *P* < 0.001) between the miRNA sequencing and RT-qPCR results, which highlights the high confidence of the results obtained using the deep-sequencing approach (Fig 7).

### The expression patterns of myomiRs (miRNA-133/-1/-206)

**Fig 8:**
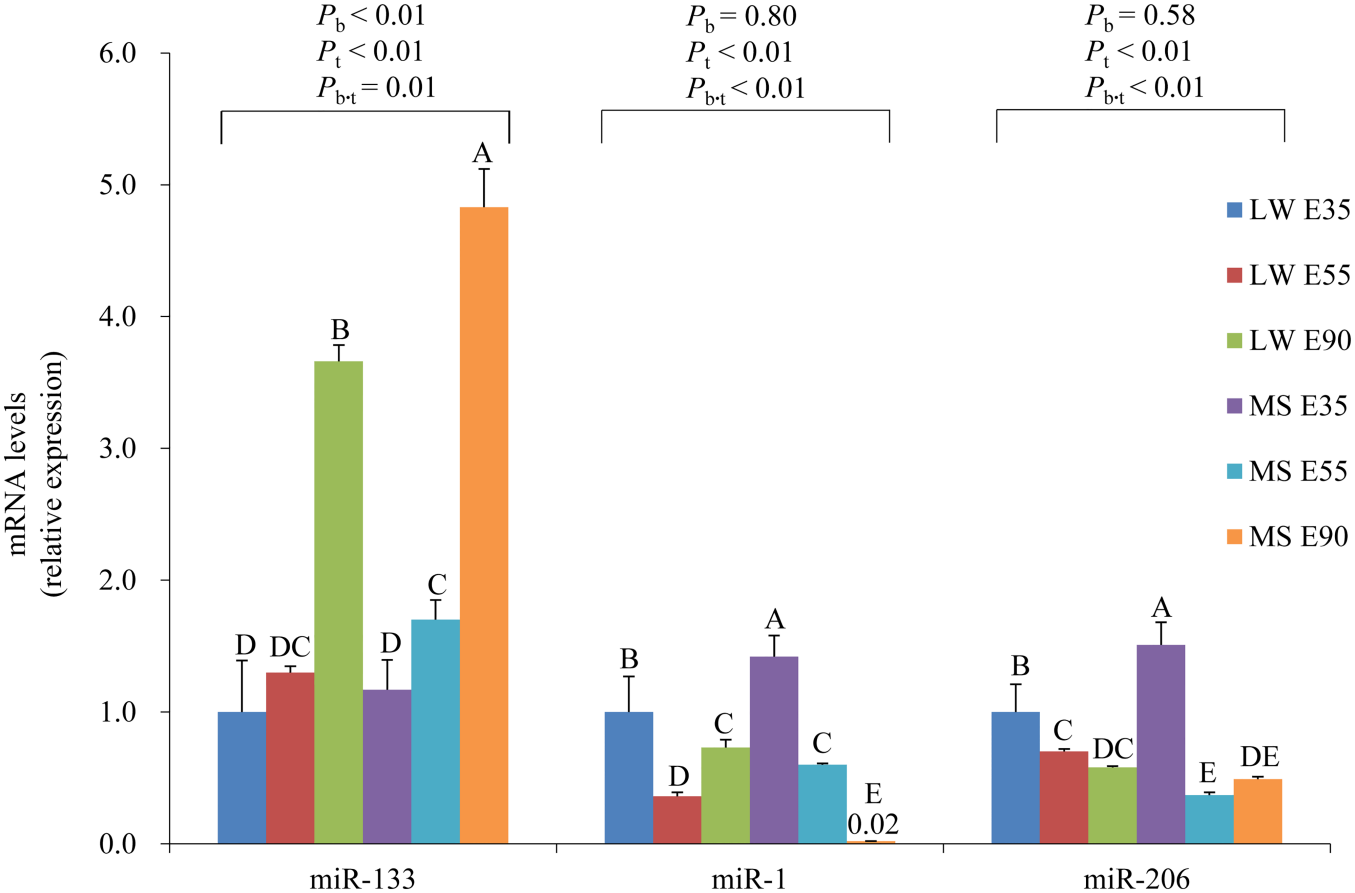
The expression levels of miR-133, miR-1 and miR-206 in Large White (LW) and Meishan (MS) pigs during skeletal muscle development at 35 to 90 dpc (E35, E55 and E90). For each miRNA, the expression level of LW pigs at 35 dpc are given as a negative control and set at 1. Two-way ANOVA (*n* = 3 per breed per time point). ‘B’ and ‘T’ mean breed and time, respectively. Values are means ± SD. Different capital letters means significant difference (*P*<0.05).

**Fig 9:**
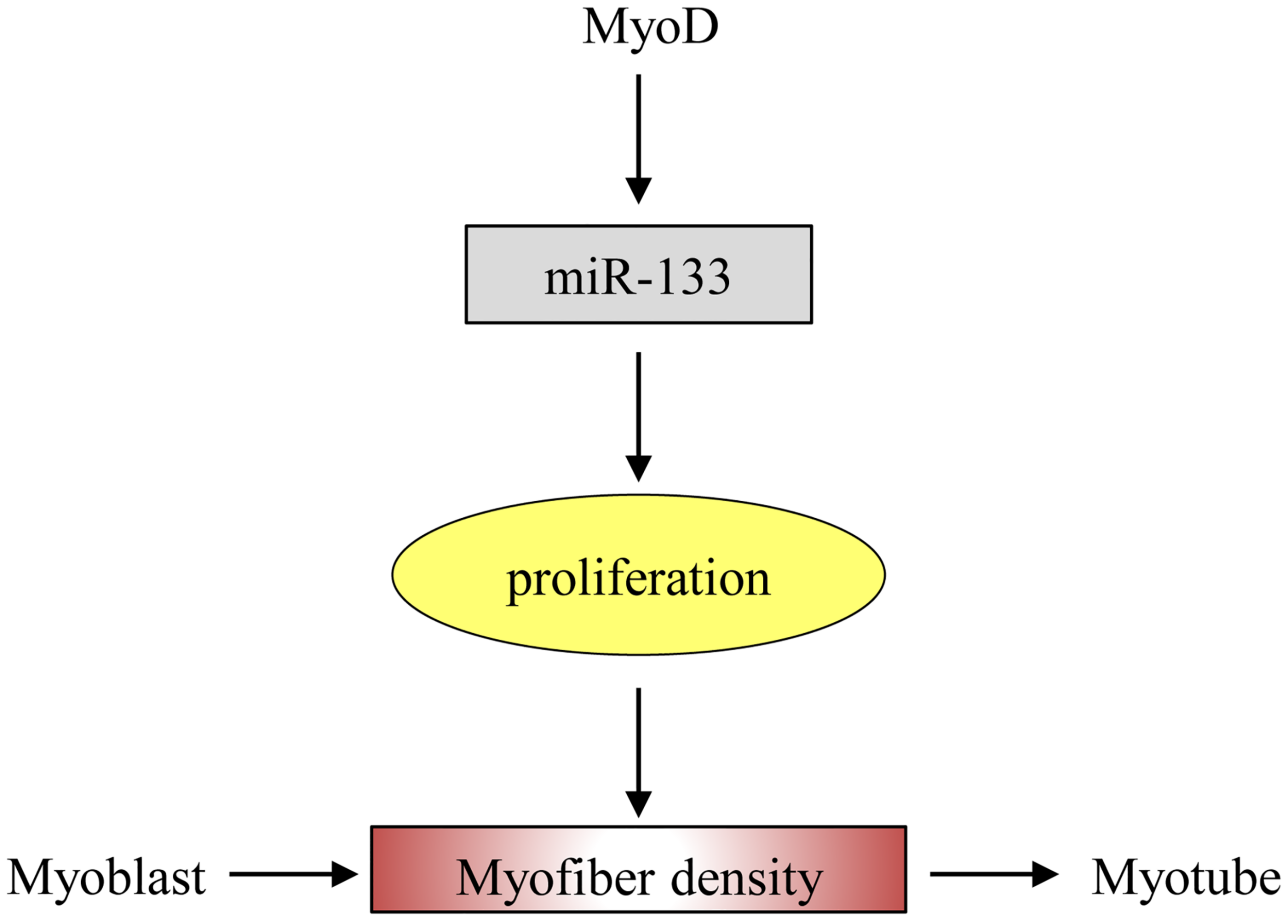
The biological function of miR-133 during skeletal muscle development. MyoD modulate muscle proliferation by regulating expression of miR-133.

The expression levels of muscle specific miRNAs, including miR-133, miR-1 and miR-206, in *longissimus dorsi* of LW and MS on pregnancy days 35, 55 and 90 were analyzed. miR-133 expression level of MS pigs was always higher than LW (*P* < 0.05), while miR-1 and miR-206 has not shown obvious expression patterns between these two breeds (Fig 8). During embryonic skeletal muscle development from 35 to 90 dpc, expression pattern of miR-133 is significantly affected by the breeds (*P*_b_ < 0.01) and development ages (*P*_t_ < 0.01). However the expression patterns of miR-1 and miR-206 from 35 to 90 dpc are similar between LW and MS, and significantly affected by developmental stages (*P*_t_ < 0.01). The role of miR-133 is enhancing myoblast proliferation, thus the upregulated miR-133 in MS may be correlated with MS’s larger myofiber density on pregnancy days 35, 55 and 90. The previous results shown that miR-133 clusters were regulated by myogenic transcription factor *MyoD* [51], and our study indicated that the expression levels of *MyoD* were significantly higher in MS than LW at 35dpc stage. All these results suggest that *MyoD* may modulate muscle proliferation by regulating expression of miR-133 (Fig 9), which is probably the main reason for different myogenesis ability between LW and MS 35dpc fetus.

## Conclusions

In conclusion, we generated miRNAome profiles of primary myofibers from LW (lean-type) and MS (obese-type) pig breeds, which show different lean growth and also different in litter size and prenatal programming. This study identified a number of DE miRNAs that were associated with porcine muscular development. This study indicate that during primary myofibers development, MS pigs show a stronger myogenesis potential than LW pigs, and miRNAs act as key regulators during primary myofibers development, and their functions were specific to different pig breeds. Some myogenesis related miRNAs (miR-133, miR-1, miR-206 and miR-148a) are highly abundant in MS pigs, while other miRNAs (let-7 family, miR-214, miR-181) highly expressed in LW. This suggests that the main miRNAs set regulating muscle development differ between two pig breeds. Further studies are needed to decipher the biological functions of these differentially expressed miRNAs. Furthermore, both primary and secondary fibers determine the size and number of final muscle fibers, while our study was focused only on the primary myofibers. However, pigs with more prenatal and postnatal developmental stages are needed in further study to uncover the underlying mechanism of refined postnatal pig skeletal muscle phenotype and meat quality. Our study provide a profound knowledge on the role of miRNAs in prenatal muscle development, which could help us to understand the different myogenesis process between LW and MS.

## Supporting information

S1 Fig Sex determination of the fetuses (PCR results for some samples). (TIF)

S2 Fig The composition of raw sequenced data. Pie chart summarizing the different classed of sequenced small RNAs in Large White (A) and Meishan pigs (B).
(TIF)

S3 Fig The length distribution of mappable reads. (TIF)

S4 Fig The differentially expressed miRNAs between Large White (LW) and Meishan (MS) pigs (*p*-value < 0.05). The codes on the legend are log_2_-transformed values.
(TIF)

S5 Fig GO enrichment scatterplot of PN-49-3p. (PDF)

S6 Fig KEGG enrichment scatterplot of PN-49-3p. (PDF)

S1 Table The primers of target and reference genes. (XLS)

S2 Table Porcine unique miRNAs. (XLS)

S3 Table Porcine known, novel and candidate miRNA. (XLS)

S4 Table The differentially expressed miRNAs. (XLS)

S5 Table The significant GO term and KEGG pathways. (XLSX)
