# Supplementary figures and images for: MicroRNA expression profiles differ between primary myofiber of lean and obese pig breeds
Source: PLoS One. 2017 Jul 31;12(7):e0181897. doi: 10.1371/journal.pone.0181897 (PMC5536276; doi:10.1371/journal.pone.0181897)

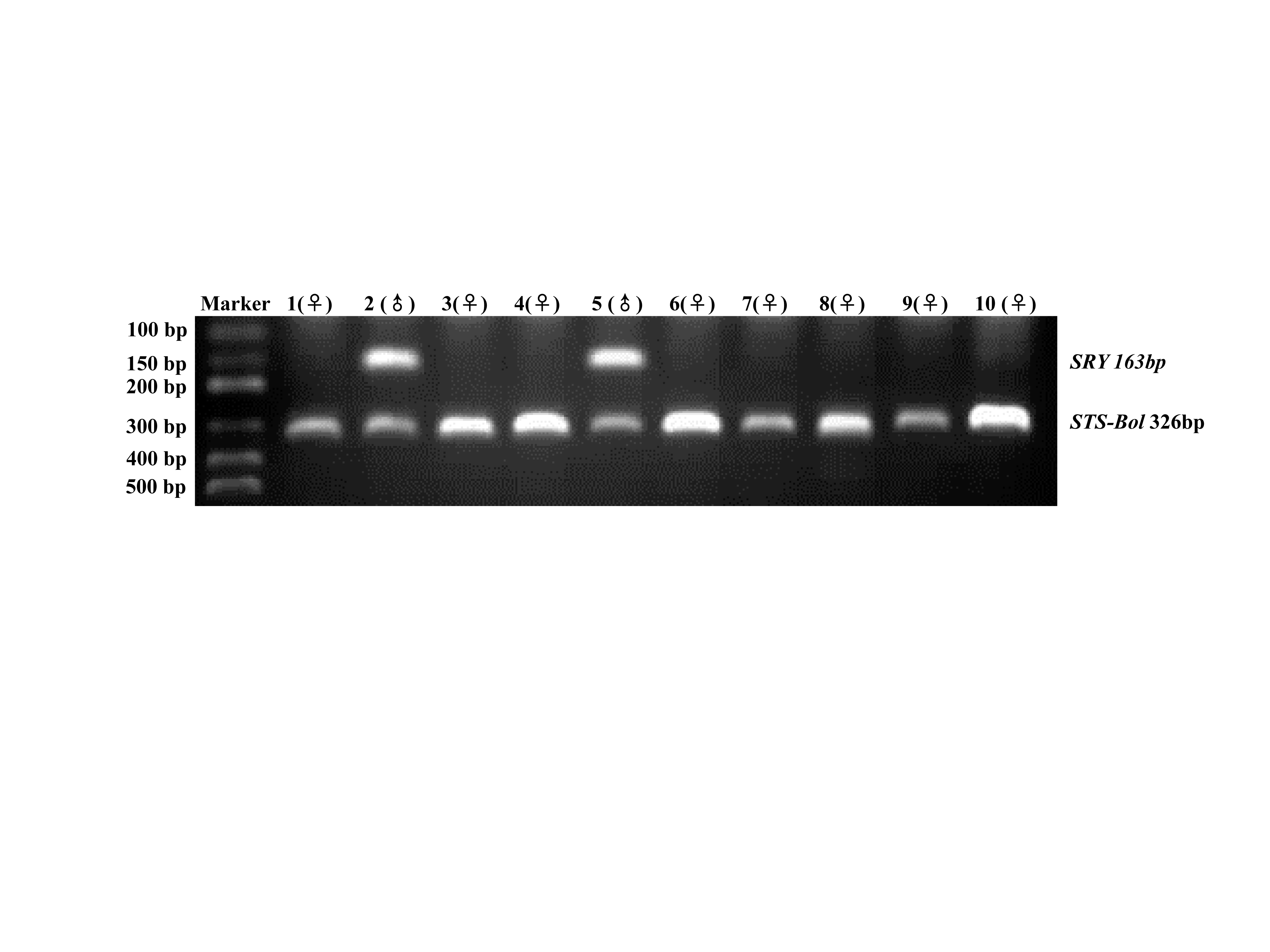

Supplement: S1 Fig — (TIF) [file pone.0181897.s001.tif]

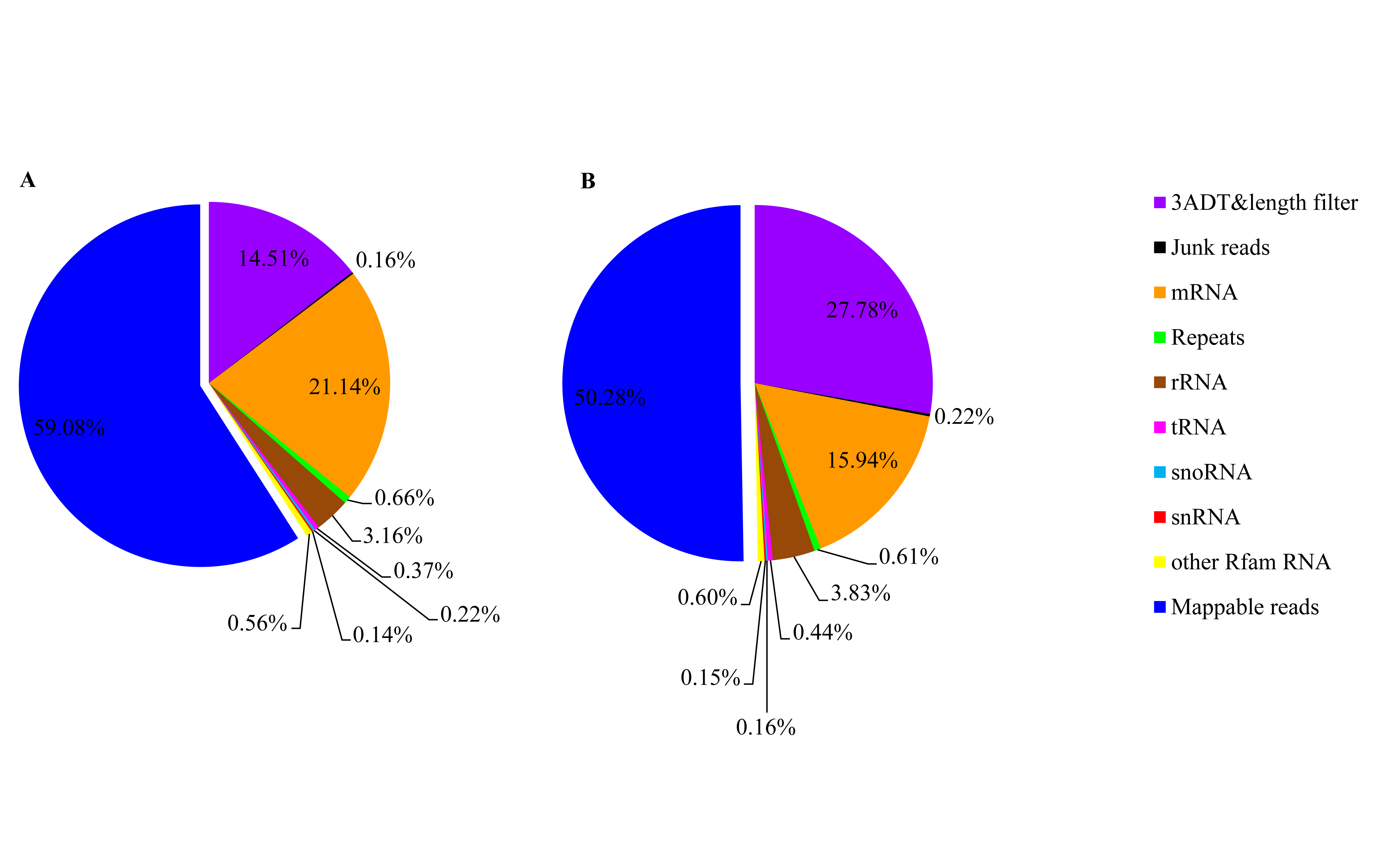

Supplement: S2 Fig — Pie chart summarizing the different classed of sequenced small RNAs in Large White (A) and Meishan pigs (B). (TIF) [file pone.0181897.s002.tif]

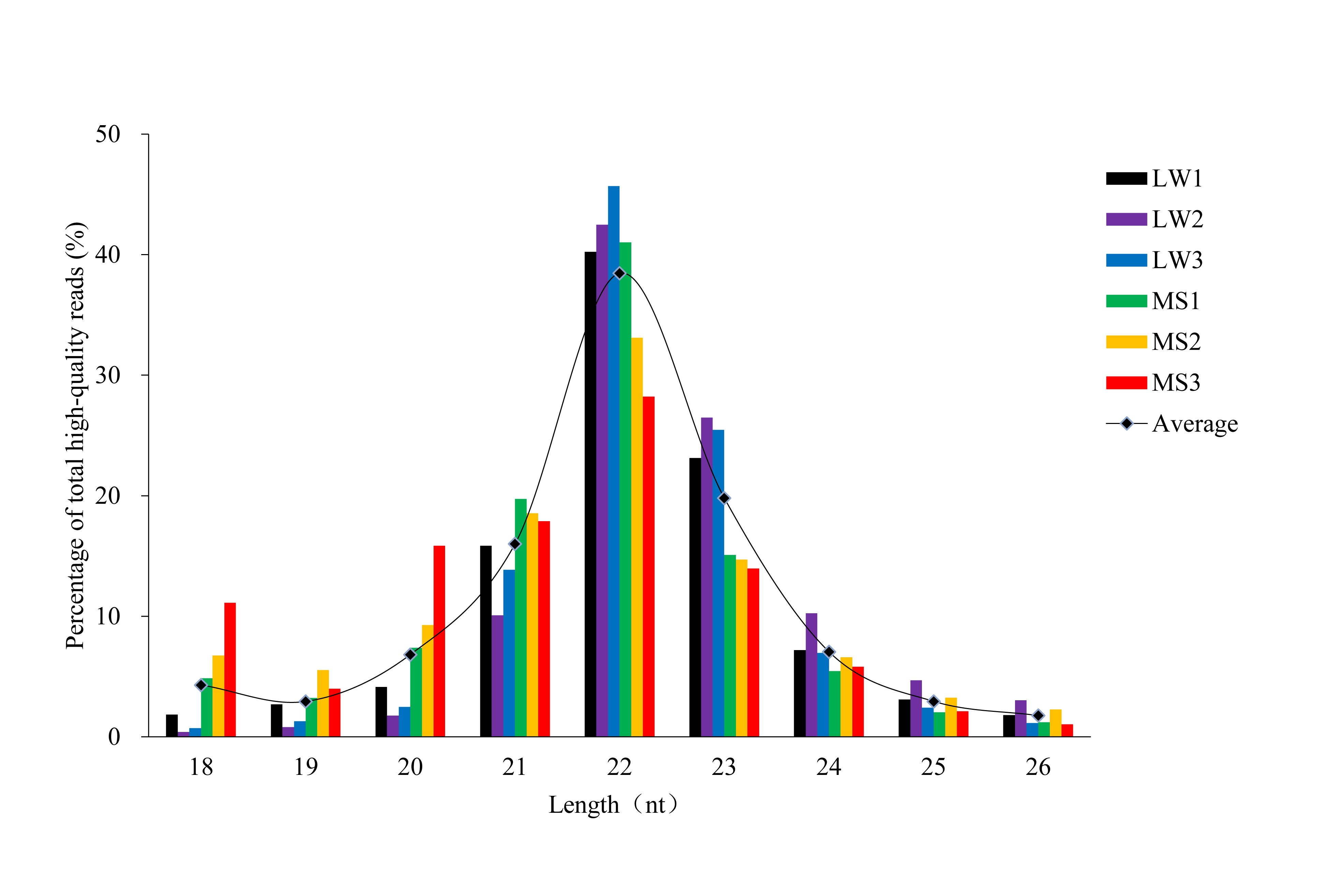

Supplement: S3 Fig — (TIF) [file pone.0181897.s003.tif]

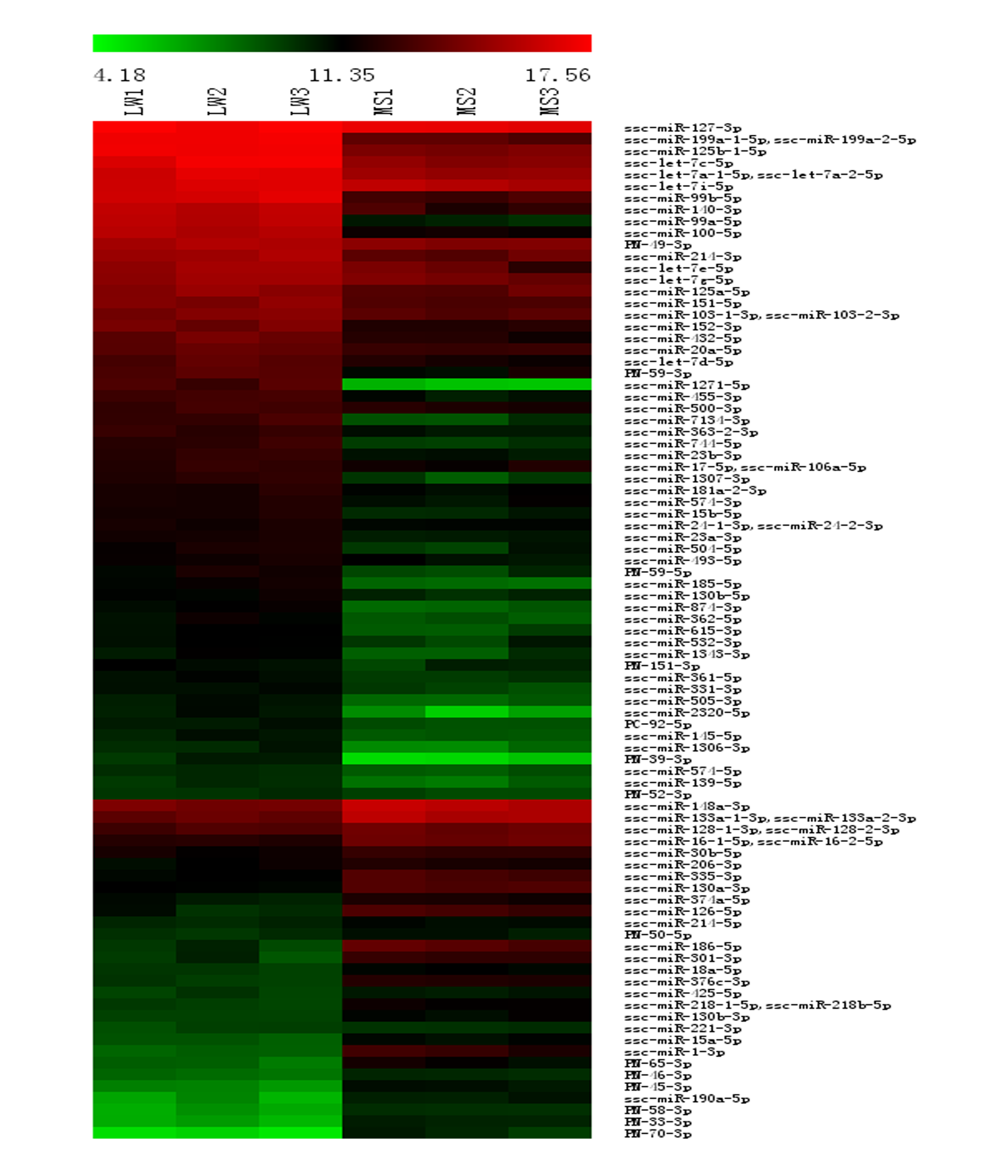

Supplement: S4 Fig — The codes on the legend are log2-transformed values. (TIF) [file pone.0181897.s004.tif]

# Statistics of GO Enrichment

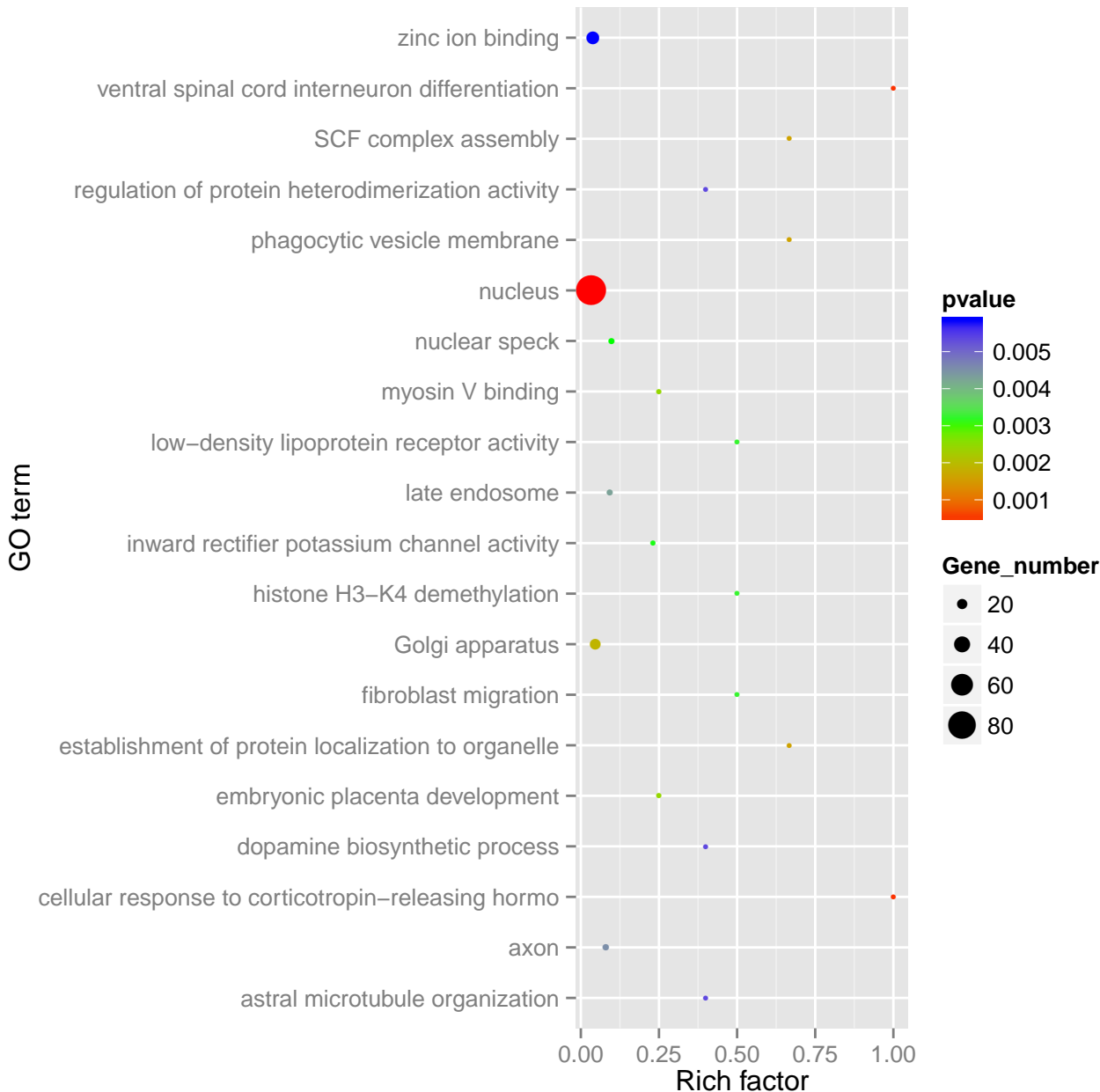

Supplement: S5 Fig — (PDF) [file pone.0181897.s005.pdf]

# Statistics of Pathway Enrichment

Pathway term

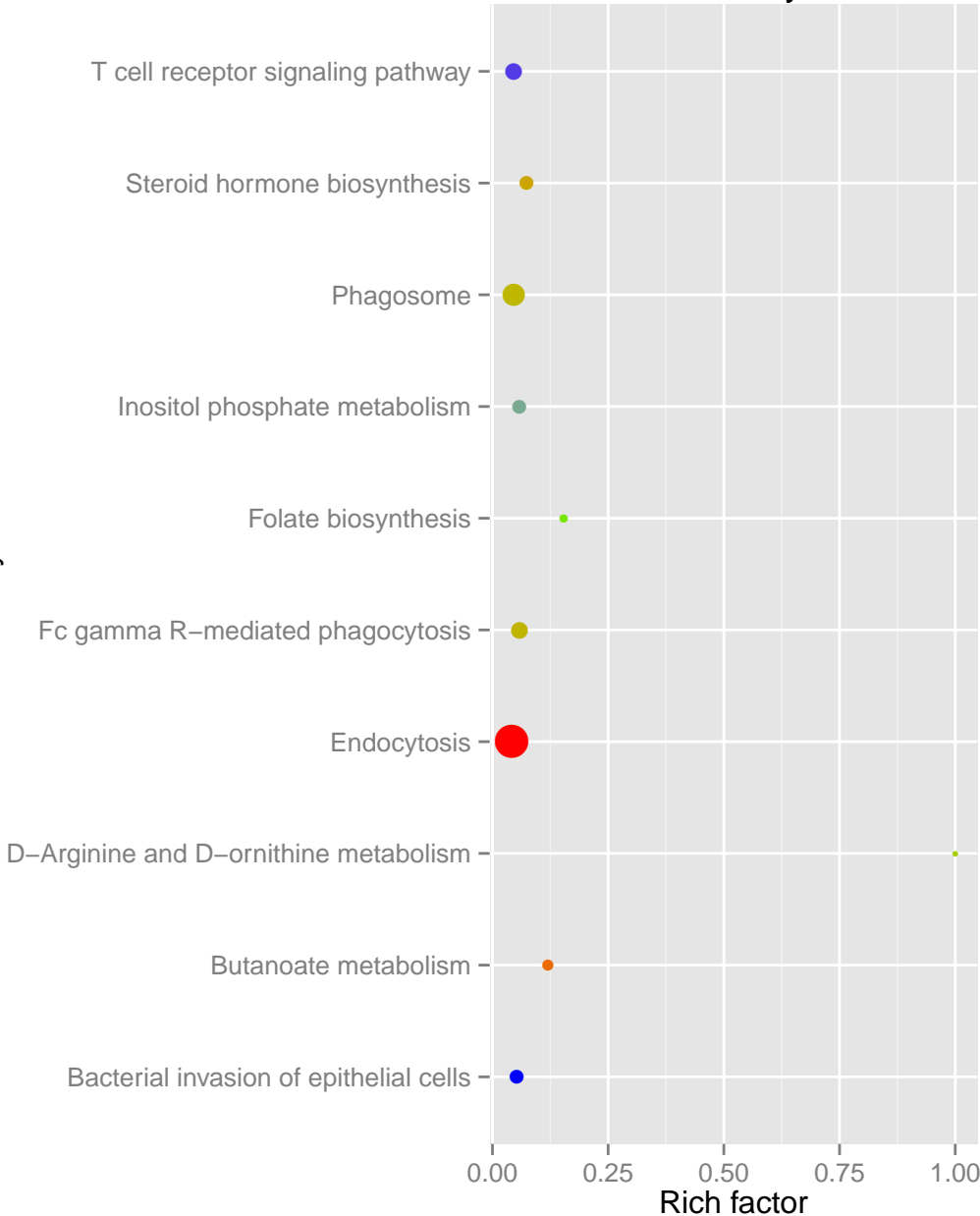

Gene\_number

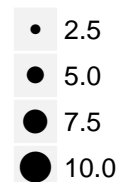

pvalue

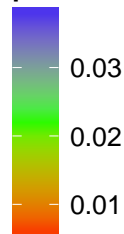

Supplement: S6 Fig — (PDF) [file pone.0181897.s006.pdf]
